# Systematic Pathologic Findings Report of *Callosciurus finlaysonii* (Horsfield, 1823) (Rodentia, Sciuridae) Squirrels from Maratea Area (South Italy) to Investigate Species-Specific Pathologies, Reliability of CO_2_ Euthanasia Method, and Possible Use as Environmental Sentinels

**DOI:** 10.3390/ani10101771

**Published:** 2020-09-30

**Authors:** Giuseppe Passantino, Massimiliano Tursi, Cristina Vercelli, Ilaria Filippi, Nicola Decaro, Antonella Tinelli, Luciana Valente, Rosa Leone, Nicola Zizzo

**Affiliations:** 1 Department of Veterinary Medicine, University of Bari “Aldo Moro”, Strada Prov.le per Casamassima Km, 3 70010 Valenzano (Bari), Italy; nicola.decaro@uniba.it (N.D.); antonella.tinelli@uniba.it (A.T.); luciana_valente@libero.it (L.V.); rosa.leone@uniba.it (R.L.); nicola.zizzo@uniba.it (N.Z.); 2 Department of Veterinary Sciences, School of Agriculture and Veterinary Medicine, University of Turin Largo Braccini 2, 10095 Grugliasco (Turin), Italy; massimiliano.tursi@unito.it; 3 Independent Researcher, 00045 Genzano di Roma (Roma), Italy; i.filippi@icloud.com

**Keywords:** systematic report, invasive species, *Callosciurus finlaysonii*, pathology, south

## Abstract

**Simple Summary:**

*Sciurus vulgaris* has been considered by the International Union for Conservation of Nature (IUCN) due to the risk of extinction caused by the invasion of the invasive species (IAS) such as *Callosciurus finlaysonii* Horsfield, 1823. This species originated from Southeast Asia and it was introduced in Italy, in 1980. These animals could cause the extinction of the autochthonous counterpart and might also represent a concern, due to the progressive and unavoidable invasion of urban areas.

**Abstract:**

The aim of the present study was to macroscopically and microscopically describe the main pathological findings occurring in this invasive species, in order to better understand the real risks for naïve animals and humans. The present study was conducted on *Callosciurus* *finlaysonii* squirrels (n = 165), captured in the Maratea area and euthanatized with CO_2_ according to a population control of invasive species of the Italian Agriculture Ministry project (ex CIPE project) and conferred to the Department of Veterinary Medicine of Bari (Italy). Macroscopic analysis demonstrated heart, lung, and liver congestion, probably due to the euthanasia method, and variable lesions of bowel, liver, and kidney. The microscopically examination showed the presence of lymphocytic infiltration in the lower layers of the bowel, suggesting enteritis. To the best of our knowledge, this is the first systemic report of gross and microscopical anatomopathological lesions in *C.* *finlaysonii,* in South Italy. The results could be useful to fill a gap of knowledge of this species in Italy.

## 1. Introduction

The red squirrel (*Sciurus vulgaris*) and the calabrian black squirrel (*Sciurus meridionalis*) are the autochthonous species of squirrel in Italy, but recently, they have been threatened by other species, which have been illegally or accidentally introduced in the Italian environment [1]. Among these alien species, it is possible to recognize the grey squirrel (*Sciurus carolinensis*), introduced in the Piedmont and Liguria regions (Northwest Italy); the siberian squirrel (*Tamias sibiricus*), released in Northern and Central Italy; and the variable squirrel (*Callosciurus finlaysonii*) [2]. This last species of squirrel belongs to the Callosciurus gene, which encodes 15 species native from Thailand and South Asia, and which was introduced in Acqui Terme (Alessandria, Piedmont region, Northwest Italy) and in Maratea (Potenza, Basilicata region, South Italy) in the mid-80s [3,4]. This species remained restricted to the Maratea area for some years, and then started to spread along the Tyrrhenian cost in both north and south directions, colonizing an area of 26 km^2^, from 2004, and becoming a serious threat to the native *S. vulgaris* [4]. A direct consequence of the introduction of alien species is the impairment of the native biodiversity, causing imbalances in the ecosystem, with negative consequences for plants and animals [5,6]. For example, it has been demonstrated that in the Maratea area, *C. finlaysonii* was responsible for bark stripping damage of Carob (*Ceratonia siliqua* L., 1753), Quercus virgiliana (*Quercus virgiliana Ten.)*, Holm (*Quercus ilex* L., 1753), and Olive (*Olea europea* L., 1753) [7,8]. The risk of extinction is very high for native species (*Sciurus vulgaris*) in Italy, and also the International Union for Conservation of Nature (IUCN) enrolled this species among those of the red list since 2013. The IUCN Red List has established an inventory of threatened species (i.e., plants, animals, and fungi) all over the world and provides information to conserve them. Its final aim is to preserve global biodiversity [9,10,11].

To avoid an ecosystem imbalance, and therefore preserve species considered to be at risk of extinction, the European Union has issued the Council Regulation (EC) 338/97 [12], implemented by Italian law in the CIPE Project 19/2004 [13] and approved by the “Istituto Superiore per la Protezione e la Ricerca Ambientale” (12/08/2009 at n. 75 AG 153998), to provide selection for alien and invasive species.

Despite the large population of *C. finlaysonii* in Maratea, very little scientific information is available and *C. finlaysonii* is currently not included officially in the EU law to control the spread of alien species [14].

The purposes of the present study were the following: (I) to provide a systematic anatomopathological description of the main findings in *C. finlaysonii* squirrels euthanized according to the National control plan, (II) to determine if the CO_2_ euthanasia method could affect the anatomo-pathological examination, and (III) to determine if this species should be considered to be a sentinel species indicative of zoonosis.

## 2. Materials and Methods

### 2.1. Capture

From 2013 to 2016, *C. finlaysonii* squirrels were captured in the Site of Community Interest (Sito di interesse comunitario, SIC) of Maratea (latitude 39° 59’ 57 N, longitude 15° 42’ 55 E). The capture was performed during the reproductive season, according to the procedures decided by local government authorities (Basilicata region, Province of Potenza, Comunità Montana del Lagonegrese and the municipality of Maratea) and respecting the national project for the control of allochthonous wildlife populations and approved by the “Istituto Superiore per la Protezione e la Ricerca Ambientale” (12/08/2009 at n. 75 AG 153998). Traps (i.e., Multicatch, Longmeadow, and Tomahawk collapsible live traps) were placed in zones, in Maratea, with high squirrel densities and areas that permitted multiple capture. Traps were checked daily and after visual recognition by an expert operator, native squirrels were released, while the alien species were humanely euthanized (i.e., inhalation of 60% CO_2_) [13].

### 2.2. Necropsies

After euthanasia, animals were frozen and sent to the Department of Veterinary Medicine of Bari (Italy) in refrigerated boxes and were preserved individually in plastic envelopes. Prior the necropsy, data about coat color, sex, and presumed age (yearlings < 1 year, subadult 1–2 years, adults > 2 years) were encoded according to the method presented by Mazzoglio et al. [5].

Necropsies were performed a few days after the arrival of the animals by an expert pathologist and included gross examination and sampling of all organs.

### 2.3. Samples Procedures

Samples were fixed in 10% buffered formalin, and finally included in paraffin. Blocks were processed with Histokinette 2000 (Reichert-Jung Gmbh, Nussloch, Germany) to obtain 3–4 µm slices, that were placed on glass and stained with Hematoxylin and Eosin staining (H. & E., Bio-Optica, Milan, Italy). Microscopically, an examination was performed using an optical microscope (Olympus BX-50, Milan, Italy). Specific colorations as periodic acid Schiff (P.A.S.), Gomori’s trichrome stain, and Grocott stain were performed when deemed appropriate using the Bio-Optica Kit method (Bio-Optica, Milan, Italy).

### 2.4. Data Collection

All the data concerning animals signalment, gross and microscopical examination were collected using Excel software (Microsoft Corporation, Redmond, WA, USA).

## 3. Results

### 3.1. Animal Signalment

**Figure 1:**
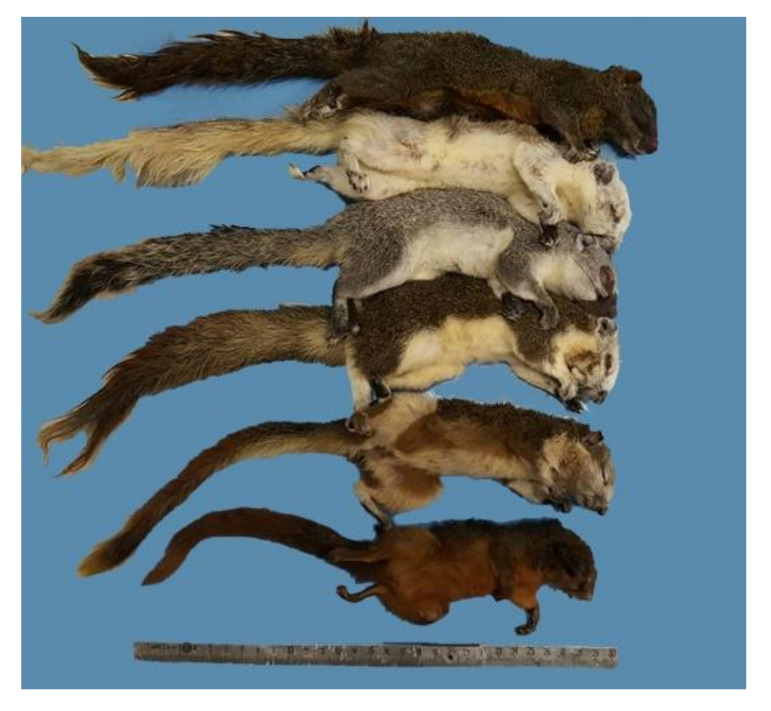
Variable squirrels (*Callosciurus finlaysonii* Horsfield, 1823).

All animals obtained were identified as *C. finlaysonii*, confirming capture selection (Figure 1). The external examination identified 123 males (51/123 yearlings, 4/123 subadults, and 68/123 adults) and 42 females (26/42 yearlings and 16/42 adults). All subjects demonstrated good body conditions and the body weight ranged from 132.2 to 263.5 g for males, and from 120.1 to 291.1 g for females, respectively.

### 3.2. External Examination

The majority of the squirrel bodies were in an excellent state of preservation due to the freezing at −20 °C within a few hours after death. Three subjects were partially decomposed. Seven out of 165 squirrels underwent tail amputations and multiple limbs, consistent with the action of predators in the entrapment phase. Fractures exposed to the limbs were found in only 4 out of 165 squirrels. There were no evident skin lesions found, except for a moderate alopecia in the nipples’ area of one female subject. Ectoparasities were not detected, except for a tick detached from the head of one squirrel and identified as a female of *Ixodes acuminatus*, also known as South rodent tick [15].

### 3.3. Organ Examination

We choose to organize the description of organs by systems and apparatus, first with gross findings, and then with microscopical details.

All subjects presented with serum-hemorrhagic effusion in the thoracoabdominal cavity; hemothorax was detected in 62 out of 165 subjects and hemoperitoneum was detected in 31 subjects out of 165. Both types of effusion were highlighted in 15 subjects.

A summary of the main findings is presented in Table 1.

#### 3.3.1. Central Nervous System

Brains and spinal cords did not show any gross or microscopically alterations.

#### 3.3.2. Lymph Nodes

**Figure 2:**
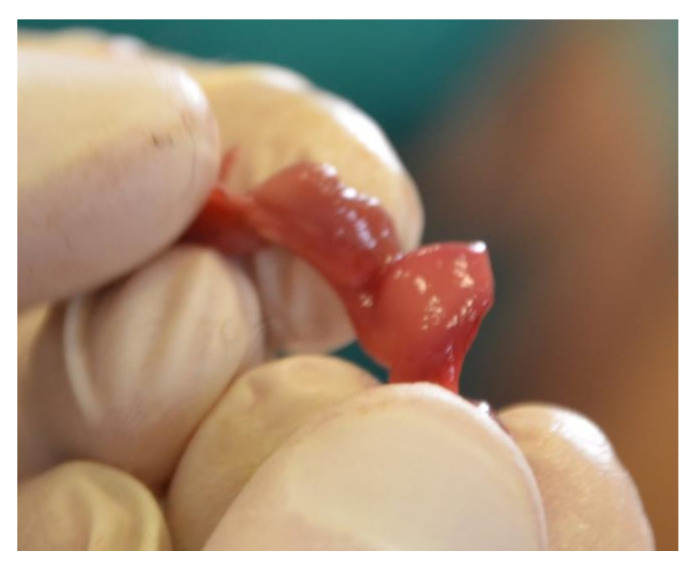
Lymph node. Pre-scapular adenomegaly.

Lymphadenopathy was found in 9 out of 165 squirrels, particularly at prescapolar lymph nodes, which showed at the histological examination diffuse and moderate to severe nonspecific follicular hyperplasia (Figure 2). In three out of nine cases severe congestion was observed.

In nine squirrels (out of 165) lymphadenomegaly was found at mesenteric lymph nodes, with bright sheared surface and soaked with serous exudate, which was considered indicative of lymphadenitis.

#### 3.3.3. Mammary Glands

**Figure 3:**
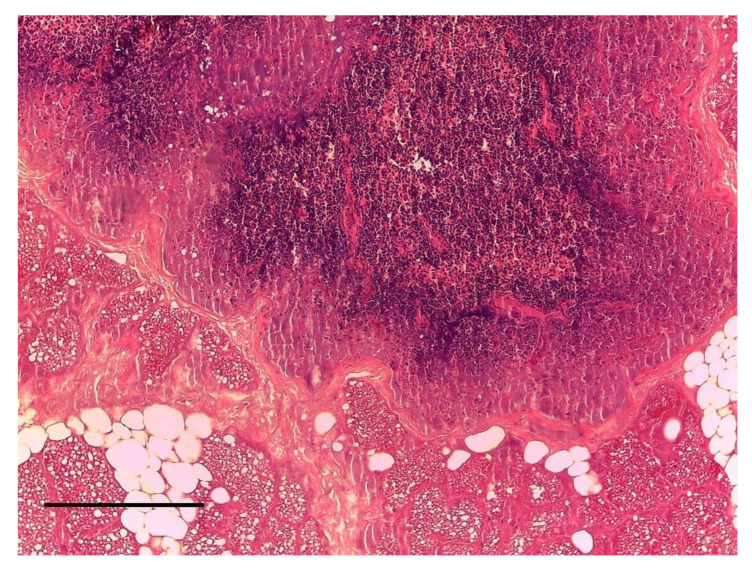
Mammary gland. Not purulent mastitis. H&E Staining, bar = 100 μm.

Enlargement of inguinal mammary glands was found in five females, associated, in four cases, with gestation. The histological examination showed a non-purulent inflammation, with multiple necrotic areas, desquamation of the glandular epithelia and foci of alveolar leukocyte infiltrate with stenosis of the intralobular ducts. All these features were associated with thickened interstitial connective tissue and the final diagnosis was not purulent mastitis (Figure 3).

#### 3.3.4. Respiratory Apparatus

**Figure 4:**
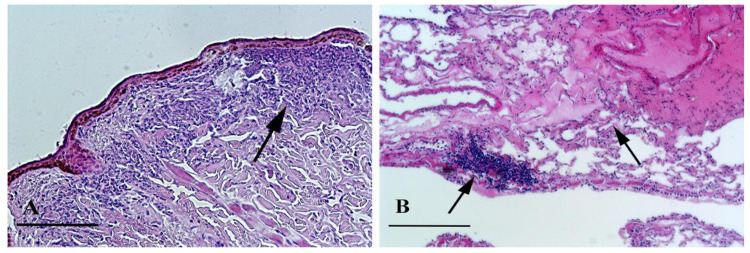
Nasal sinuses. (**A**) Inflammatory infiltrator (arrow). H&E staining, bar = 100 μm; (**B**) Inflammatory infiltrator and edema (arrows). H&E staining, bar = 100 μm.

Nasal sinuses of four squirrels (out of 165) demonstrated the presence of catarrhal exudates and edematous rhino-sinus mucosa. Microscopically, lamina propria was infiltrated by leukocyte exudate, with hyperemic vessels (Figure 4).

Lung lesions were found in 142 out of 165 cases and were mainly attributable atelectasis, emphysema, edema, fibrinous pneumonia, and congestion (details reported in the Table 2).

#### 3.3.5. Cardiovascular Apparatus

The examination of main arteries and veins did not show alterations.

**Figure 5:**
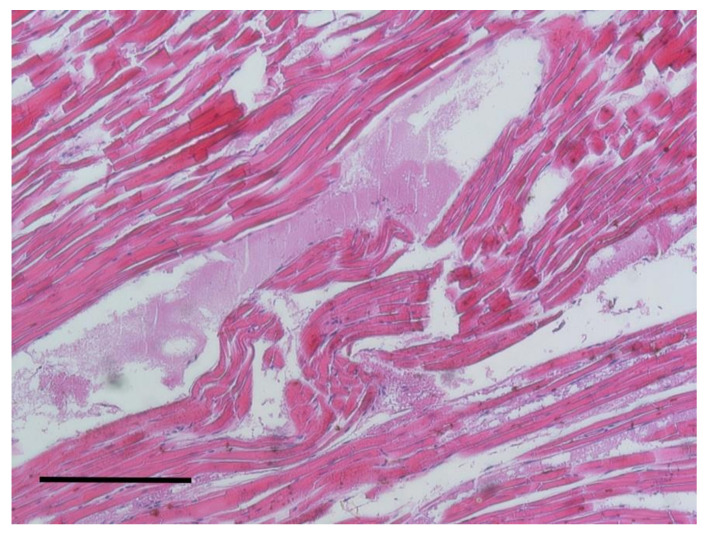
Heart. Disruption, fragmentation of muscle fibers and edema. H&E staining, bar = 100 μm.

The gross examination of hearts highlighted the presence of an enlargement in the majority of the subjects. Right ventricles were enlarged with a decreasing consistency. Histologically, a disordered arrangement of myofibers with fragmentation and interstitial edema was observed (Figure 5).

#### 3.3.6. Abdominal Organs

**Figure 6:**
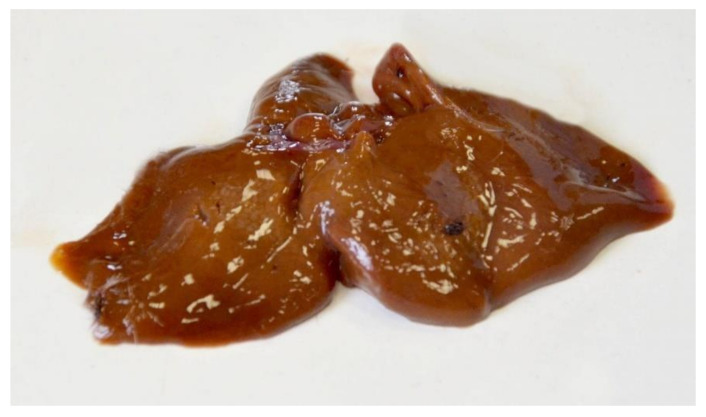
Liver. Appearance of yellowish color. Hepatosis.

Hepatic lesions were found in 142 out of 165 squirrels. In 23 cases (out of 142), a microscopical examination identified a serous hepatitis, characterized by hepatomegaly, thickening of the capsule, and rounded edges. Parenchyma soaked in serous exudate at the cutting surface. Another 88 cases (out of 142) were identified as hepatosis, in which the consistency was friable and both external and cutting surface were grayish-yellowish. In the other 31 cases (out of 142), it was possible to appreciate a hepatic congestion with hepatomegaly, rounded margins, and the parenchyma were dark red in color and with blood shedding at cutting surface. Histologically, we observed the presence of dilated sinusoids filled with erythrocytes and peripheral lobule cells in the anoxic phase, characterized by vacuolar formations of slightly pink color or optically empty. These two conditions were assigned to vacuolar degeneration and fatty degeneration, respectively (Figure 6).

The gross examination of spleens demonstrated the congestion in 54 out of 165 subjects, with an increased in volume and rounded margins. No cases showed capsule abnormalities. At the cutting surface, a red-blood spillage and well-retained pulp was highlighted. Histologically, the congestion was confirmed by the identification of flesh and sinuses full of numerous erythrocytes which dilated the entire structure.

**Figure 7:**
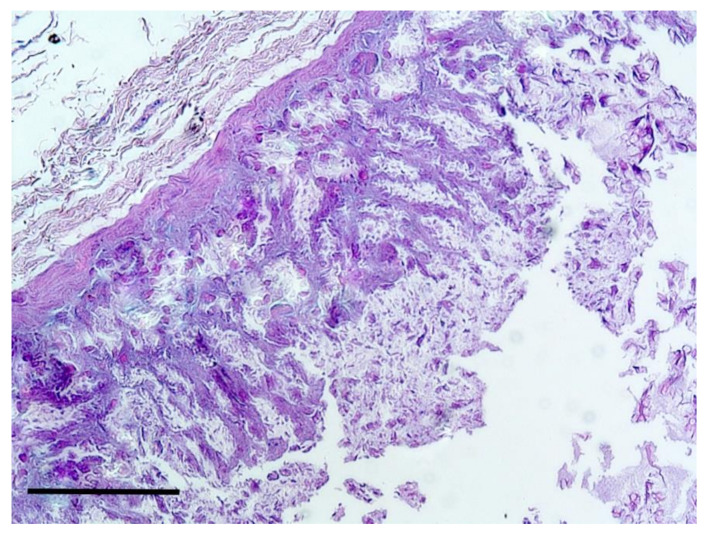
Stomach. Exfoliation of the mucosa with presence of catarrhal exudate mixed with inflammatory cells. H&E staining, bar = 100 μm.

The gross examination of the digestive apparatus showed the presence of hyperemic and thickening of the gastric mucosa with a flowing exudate (Figure 7). This condition was recognized in 90 cases out of 165. In same subjects, enteric mucosa was sprinkled with abundant catarrhal exudate, streamlined and yellowish color of the bowel mucosa. No ulcers or hyperemic areas were found but intestinal content was indicative of diarrhea. Histologically, an exfoliation of the epithelium, with mucinous material outside the intestinal crypts, was highlighted. In the deepest parts of the mucosa, we recognized a mild inflammation with lymphocytic infiltration.

#### 3.3.7. Urinary Tract

The gross examination of the urinary tract encoded kidneys, ureters, and bladder.

**Figure 8:**
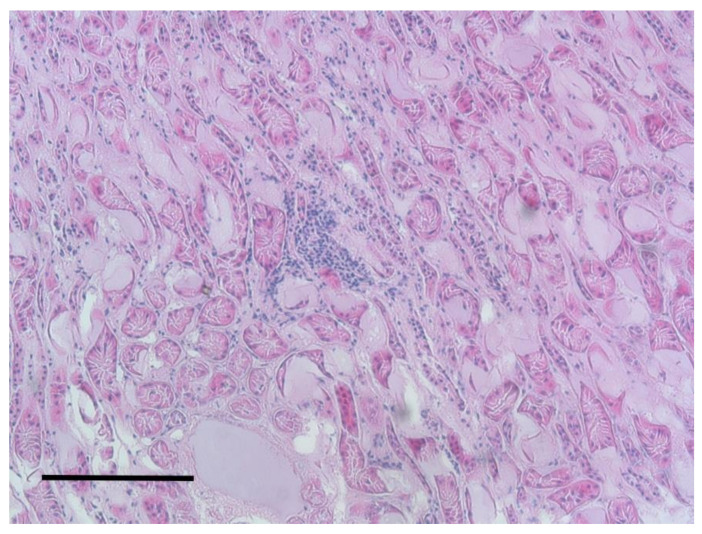
Kidney. Peritubular lymph-plasmacellular inflammation. H&E staining, bar = 100 μm.

Renal lesions were found in 30 squirrels out of 165; 15 of them were identified as nephritis with shiny appearance in cut surface and ectasia of cortical vessels. The other 15 cases were recognized as nephrosis with a predominant greyish aspect at the cut surface. Microscopically, the proximal convoluted tubules had dense and coagulated protoplasm of epithelial cells. Small necrotic areas were identified in the lower parts of the nephron with lymphoid interstitial infiltration (Figure 8).

No dilatations or stenosis were identified in ureters. The gross and microscopic examination of bladders and ovaries did not show abnormalities.

#### 3.3.8. Reproductive System

To examine the reproductive system, uterus and ovaries of all female subjects, and testicles and penis of all male subjects, were checked, respectively.

An increase in prostate volume was found in five out of 123 males.

**Figure 9:**
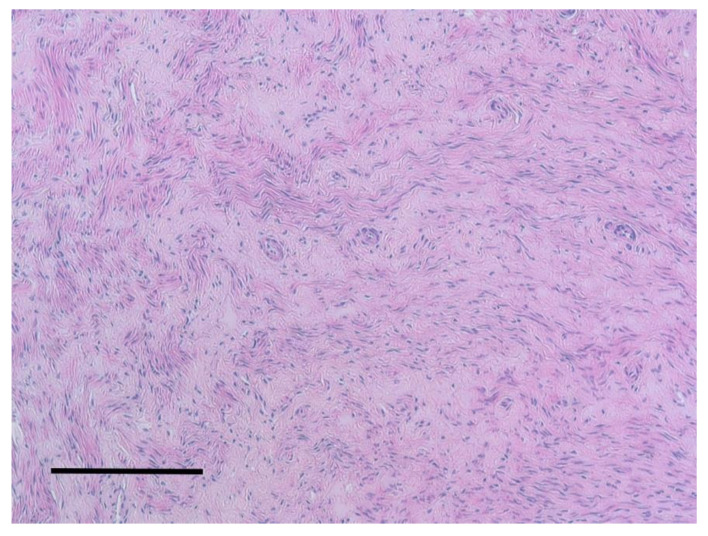
Uterus. Leiomyoma. Cells resemble smooth muscle, are relatively uniform and had no mitoses. H&E staining, bar = 100 μm.

**Figure 10:**
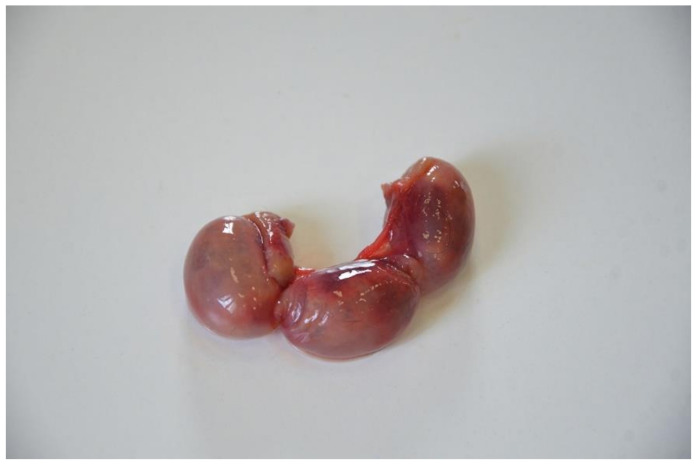
Uterus. Pregnant uterus.

In 10 cases, an increased volume in the uterus, hyperemic mucosa, and an abundant pinkish liquid or catarrhal exudate content were highlighted. All these organs showed a thickened wall and a bright red colored mucosa and muscular layer. Microscopically, three cases showed signs of edema and the presence of erythrocytes, while in seven cases, neutrophil infiltration was observed in the mucosa and glands (endometritis). No sign affected the external serous layer. In one case out of 42, the gross examination revealed an irregular appearance, correspondent to the presence of a firm mass, compact with a lighter color than the surrounding tissue. Microscopically, the smooth muscle layer appeared quite distinct from the surrounding tissue and with a pattern characterized by mature cells with the absence of mitotic figures at high power field (HPF) (Figure 9). The smooth muscle fibers were organized in a whirling pattern. The surrounding healthy tissue was compressed by the mass. This led to the diagnosis of uterine leiomyoma. Four squirrels out of 42 were identified as pregnant (Figure 10).

## 4. Discussion

To the best of our knowledge, this is the first systemic description of anatomo-pathological findings in *C. finlaysonii* squirrels in the Maratea area.

The primary lesions were observed in lungs (86%), liver (86%), stomach-intestine (54.5%), and kidney (18%). Moreover, many lesions were found in adult (82%) subjects rather than in yearlings (18%). Even if this could sound anecdotical, it is coherent. Some pathologies require a few times to demonstrate signs and symptoms.

Serum-hemorrhagic effusion was found in the thoracoabdominal cavity probably, related to thawing and to the action of CO_2_. Hemothorax and hemoperitoneum was detected in the absence of evident traumatic injuries to the cavities or organs. The congestion of the lungs and of other parenchymatous organs, as well as the bright red color of the blood might be due to the euthanasia method using CO_2_ [16,17]. An investigation conducted on mice, demonstrated that the CO_2_ euthanasia method can cause mainly perivascular and peribronchiolar edema, and less congestion and hemorrhage [18]. The same investigation also considered the isoflurane inhalation and the intra-peritoneal injection of barbiturics as alternative euthanasia methods, and these two techniques also caused less but similar lung lesions. Nevertheless, in the present study, it was possible to appreciate degenerative or inflammatory lung lesions characterized by the presence of inflammatory cells, fibrin, emphysema, or atelectasis and lymph node reactivity.

The occurrence of lymphadenomegaly and gastroenteric phlogosis, often accompanied by hepatic lesions could be indicative of malabsorption phenomena. It must be born in mind that *C. finlaysonii* is an alien species that could not find the same environmental and dietary conditions of the native habitat. In addition, hepatic, gastrointestinal, and renal lesions may be related to a metabolic disorder. This species is considered to be an important seed consumer and seed dispersal agent and also a frequent predator of birds’ nests [8,19,20]. Squirrels, introduced in Italy, feed mainly on seeds, fruits, buds, flowers and, occasionally, on insects, switching from one resource to another according to seasonal availability. In particular, during autumn and winter times, *C. finlaysonii* has the habit of staying on trees, discarding the bark (bark-stripping damage), and then licking the sap arising from the underling parts, creating considerable damage to trees [7,21,22]. Moreover, in the Maratea region, the majority of trees are oaks rich in tanninis, whose effects are not proven in *C. finlaysonii* squirrels. Large quantities of tannins could cause serious gastrointestinal disorders [23,24]. According to this, it is reasonable to suppose that the gastrointestinal lesions could be caused by a dietary imbalance, even considering the longest winter period in Italy as compared with their native countries.

The four pregnant females did not show any particular lesions except hepatic degeneration, probably caused by both diet and pregnancy. 

In all the examined subjects, cardiac damage was identified. The fibers were degenerate and infiltrated by serous exudates. In our opinion, this condition could be related to interaction of several factors. The physiology of these animals encoded a very high heart rate (500 beats per minute) and no studies have been performed to investigate the normal heart features of squirrels. Moreover, the euthanasia method can cause an increasing heart rate and stress [18,25]. The impact of stress is difficult to assess but several studies investigated this according to the invasion of alien squirrel species [26].

The last aim of the study was to evaluate the usefulness of these animals as a potential sentinel of human disease [27]. A microbiological analysis was conducted to isolate and identify contaminants that were not taken into consideration (data not shown). A very low incidence of neoplasms was found in *C. finlaysonii* squirrels examined in the present project. In contrast to the report in a similar study conducted about red squirrels (*Sciurus vulgaris*) in Great Britain, the results showed a high presence of neoplasia that was related to the fact that red squirrels lived in urban habitats and the pollution could increase the incidence of cancer [28].

## 5. Conclusions

This is the first study to provide a systemic description of the most common anatomopathological lesions found in *C. finlaysonii* squirrels of the Maratea area. Furthermore, intestinal, renal, and hepatic lesions were often associated, and these data seem to suggest the hypothesis of a metabolic disorder caused by a diet not suitable for this alien species. There were only a few lesions that could be associated with the euthanasia method and freezing process and that did not significantly affect the reliability of the systematic anatomo-pathological investigation. Further studies would be necessary to understand both physiological and pathological heart conditions of squirrels.

## Figures and Tables

**Table 1:** Percentage of the lesion retrieved.

Organs/Total Examined (%)	Lesions	Lesion/Affected Organs (%)
Lymph node 9/165 (5%)	Acute serous lymphadenitis	9/9 (100%)
Lung 142/165 (86%)	Atelectasis	5/142 (3,5%)
	Emphysema	11/142 (7,7%)
	Edema	26/142 (18%)
	Fibrinous pneumonia	73/142 (51%)
	Congestion	95/142 (67%)
Liver 142/165 (86%)	Hepatitis	23/142 (16%)
	Hepatosis	88/142 (62%)
	Hepatic congestion	31/142 (22%)
Kidney 30/165 (18%)	Nephritis	15/30 (50%)
	Nephrosis	15/30 (50%)
Uterus 11/42 (26,2%)	Endometritis	7/42 (16,6%)
	Edema	3/42 (7,1%)
	Congestion	10/11 (90,9%)
	Neoplasia	1/11 (9,1%)
Prostate 5/123 (4,1%)	Hyperplasia	5/5 (100%)
Heart165/165 (100%)	Degenerative processes	165/165 (100)
	Right heart collapse	69/165 (41,8%)
Alimentary tract 90/165 (54,5%)	Catarrhal gastritis	90/90 (100%)
	Catarrhal enteritis	90/90 (100%)
Mammary Gland 5/42 (11,9%)	Hypertrophy	5/5 (100%)
	Mastitis	5/5 (100%)
Spleen 54/165 (33%)	Congestion	54/54 (100%)
Thoracoabdominal Cavity 93/165 (56%)	Haemoperitoneum	31/93 (33,3%)
	Haemothorax	62/93 (66,6%)
	Both	15/93 (16,1%)

**Table 2:** The table summarize the main findings in lungs.

Lesion	Cases	Macroscopically	Histologically
Atelectasis	5/142	Dark red color areas and increase consistency	Alveolar lumen disappeared due to collapse of the walls. The alveolar septa and bronchiolar lumens filled with blood, with light interstitial edema;
Emphysema	11/142	Increased volume, pale pink color and increased crackling	dilated alveolar walls with loss of continuity of the wall of the alveoli due to breakage of the walls;
Edema	26/142 interstitial septums	Increased volume, rounded margins, shiny and soaked appearance	pink colored fluid in the alveoli with epithelium and pneumocytes II type increased in number, edematous
Fibrinous pneumonia	73/142	Marbled appearance areas, rounded margins and increase consistency of apical lobes red hepatization)	alveolar lumens with desquamated epithelium filled with fibrin and erythrocytes, neutrophils, and lymphocytes. Ectasia of blood and lymphatic vessels;
Congestion	95/142	Bright red color, leakage of uncoagulated blood from the cutting surface	parenchyma with dilated capillary network for accumulation of RBC and rare phenomena of erythrophagocytosis

## Data Availability

All data generated or analyzed during this study are included in this published article. If any additional material used or analyzed during the current study is required, these are available from the corresponding author on reasonable request.

## Abbreviations

*C. finlaysonii*	*Callosciurus finlaysonii*
IUCN	International Union for Conservation of Nature
CO_2_	Carbon dioxide;
*S. vulgaris*	*Sciurus vulgaris*
CIPE	Comitato interministeriale per la programmazione economica
ISPRA	Istituto Superiore per la Protezione e la Ricerca Ambientale
SIC	Siti di interesse comunitario
H. & E.	Hematoxylin eosin stain
P.A.S.	Periodic acid Schiff stain
*I. acuminatus*	*Ixodes acuminatus*
HPF	High power field
